# Correlation of Ultrasound, Hysteroscopy, and Histology in Postmenopausal Women: A Five-Year Retrospective Observational Study in Dubai

**DOI:** 10.7759/cureus.106068

**Published:** 2026-03-29

**Authors:** Ayesha Salman, Zenab Yusuf Tambawala, Shabnam Saquib, Nighat Fatima, Seema Waheed, Lama K Hamza

**Affiliations:** 1 Obstetrics and Gynecology, Dubai Hospital, Dubai, ARE

**Keywords:** diagnostic hysteroscopy, endometrial carcinomas, endometrial hyperplasia, evaluation of postmenopausal bleeding, postmenopausal bleeding, transvaginal ultrasound scan

## Abstract

Introduction

This study investigates the relationship between endometrial thickness measured by ultrasound, hysteroscopic findings, and histological diagnosis of endometrial pathology. It aims to evaluate the diagnostic efficacy of ultrasound and hysteroscopy in detecting endometrial malignancy in postmenopausal women and in high-risk groups such as breast cancer patients on tamoxifen.

Methods

This retrospective observational study was conducted at Dubai Hospital between January 2018 and December 2022. Ethical approval was obtained from the Dubai Scientific Research Ethics Committee. Histopathology was used as the reference (gold) standard for all calculations of sensitivity, specificity, positive predictive value (PPV), and negative predictive value (NPV).

Results

A total of 168 post-menopausal patients were analyzed. The mean age of the participants was 59.39 ± 7.825 years (range: 44-86 years). The mean body mass index was 34.60 ± 7.60 kg/m², with 73.1 % of participants obese and 19.6% morbidly obese. The mean duration of menopause was 8.220±6.66 years. A total of 132 (78.6%) participants presented with postmenopausal bleeding. The mean endometrial thickness was 11.084 ± 6.19 mm. The frequency of endometrial cancer increased with endometrial thickness, with malignancy identified in 0 of 19 cases (<5 mm), 3 of 72 cases (4.2%; 5-10 mm), 1 of 46 cases (2.2%; 10.1-15 mm), and 1 of 20 cases (5.0%; 15.1-20 mm); notably, the highest malignancy rate was observed in the >20 mm group, where 5 of 11 cases (45.5%) were malignant, although this subgroup represented the smallest proportion of the cohort (6.5%). Benign polyps (90; 53.5%) were the most common histopathological findings. Endometrial carcinoma was diagnosed in 10 (6.0%) of cases and endometrial hyperplasia in 11 (6.5%) cases. At the threshold of≤ 5 mm, ultrasound had a sensitivity of 100%, specificity of 12%, PPV of 67%, and NPV of 100%. Hysteroscopy showed sensitivity of 80%, specificity of 96.2%, PPV of 57.1%, and NPV of 98.7%.

In post-menopausal breast cancer patients, the mean endometrial thickness was 11.361 ± 5.079 mm. No cases of endometrial cancer were identified in the breast cancer cohort.

Conclusion

Transvaginal ultrasound is a reliable first-line investigation for postmenopausal bleeding, with high sensitivity but limited specificity. Hysteroscopy provides high specificity and the ability to obtain biopsies.

## Introduction

Postmenopausal bleeding (PMB), clinically defined as any vaginal bleeding occurring 12 months or more following the final menstrual period [1], represents a common yet clinically significant presentation, affecting approximately 10-15% of postmenopausal women [2,3]. Although the majority of cases arise from benign etiologies, most notably endometrial atrophy and endometrial polyps, PMB may also herald more concerning pathologies, including endometrial hyperplasia and endometrial carcinoma.

In contrast to ovarian cancer, endometrial cancer frequently presents at an early stage, thereby enabling curative intervention through surgical hysterectomy. Early detection of endometrial malignancy is paramount, as patients diagnosed with localized disease demonstrate a favourable five-year survival rate of approximately 95%. However, prognosis deteriorates significantly with disease progression: 5-year survival declines to approximately 70% in cases with regional metastasis and further diminishes to 20% when distant metastatic spread has occurred [4].

For postmenopausal women presenting with vaginal bleeding, clinical guidelines recommend prompt evaluation. Both the American College of Obstetricians and Gynaecologists (ACOG) [5] and the Society of Radiologists in Ultrasound (SRU) [6] endorse either endometrial sampling or transvaginal ultrasound (TVUS) as appropriate first-line diagnostic modalities.

TVUS provides a reliable assessment of endometrial thickness and morphology, thereby facilitating the identification of women with postmenopausal bleeding who exhibit a thin endometrium (≤4 mm) and demonstrate a low probability of harbouring endometrial malignancy [7-9]. Accumulating evidence suggests that endometrial thickness (ET) exceeding 4 mm warrants further evaluation for potential endometrial pathology, as increased endometrial thickness demonstrates a positive correlation with higher rates of endometrial hyperplasia [2,3,10]. Thresholds of ET <4 mm, ≤ 4 mm, <5 mm, and ≤5 mm have been used in various studies; in our study, we have kept it as ≤5 mm.

Compared with conventional diagnostic methods, such as dilation and curettage (D&C), hysteroscopy enables the direct visualization of focal endometrial abnormalities and allows for targeted tissue sampling under direct vision. Hysteroscopic examination combined with histopathological analysis serves to establish a definitive diagnosis and exclude malignancy. In a systematic review by Clark et al., hysteroscopy demonstrated high diagnostic accuracy for endometrial carcinoma; however, its accuracy was only moderate for detecting the broader spectrum of endometrial disease, defined as carcinoma and/or hyperplasia [11]. These findings underscore the necessity of comprehensive diagnostic evaluation, particularly in high-risk populations. Clinical risk factors for endometrial cancer extend beyond the traditional triad of advanced age, obesity, and unopposed oestrogen exposure. Additional metabolic and endocrine comorbidities, including PCOS, type 2 diabetes mellitus, and hypertension, should be systematically considered during the evaluation of postmenopausal bleeding [9].

Tamoxifen is widely employed as adjuvant hormonal therapy in the management of oestrogen receptor (ER)-positive breast cancer. While tamoxifen functions as an ER antagonist in breast tissue, it paradoxically exerts ER agonist activity in the endometrium, thereby conferring an elevated risk of various endometrial pathologies [12,13]. The incidence of these complications increases substantially with prolonged tamoxifen exposure [14].

Ultrasonographic evaluation of women receiving tamoxifen therapy frequently demonstrates cystic endometrial thickening. However, this sonographic appearance exhibits poor correlation with hysteroscopic findings, as the endometrium appears atrophic rather than hyperplastic in 50-90% of cases upon direct visualisation. Transvaginal ultrasonography (TVUS) demonstrates significant limitations in accurately characterizing tamoxifen-associated endometrial changes, yielding false-positive results in 46-56% of examinations [12,13]. Given the inherent limitations of TVUS as a standalone diagnostic modality in this population, additional diagnostic procedures, including hysteroscopy with directed biopsy, are warranted for definitive evaluation.

Given these diagnostic challenges and the limitations of current evidence, particularly in high-risk populations, this study aims to evaluate the concordance between endometrial thickness, hysteroscopic findings, and histopathology in postmenopausal women; to determine the optimal endometrial thickness threshold for predicting malignancy in our population; to assess the diagnostic performance of TVUS and hysteroscopy against the gold standard of histology; and to specifically examine these relationships in the subset of breast cancer patients on tamoxifen therapy.

## Materials and methods

This retrospective observational study was conducted at Dubai Hospital, a 625-bed tertiary care facility, over a five-year period spanning January 2018 to December 2022. Ethical approval was secured from the Dubai Scientific Research Ethics Committee (DSREC-09/2023_10), ensuring adherence to established ethical standards for clinical research. The study population comprised postmenopausal women aged 40 years and older who underwent diagnostic hysteroscopy for endometrial evaluation during the study period. Patients were excluded from the study if they had a prior diagnosis of endometrial or cervical malignancy, the presence of cervical stenosis precluding adequate endometrial sampling, the absence of an ultrasound report, or incomplete clinical or histopathological records.

The primary objectives were to evaluate the concordance between endometrial thickness measurements, hysteroscopic findings, and histopathological diagnosis and to assess the diagnostic performance of ultrasonography and hysteroscopy in detecting endometrial pathology among postmenopausal women. Secondary objectives included examining the correlation between endometrial thickness and histological findings in patients with a history of breast cancer receiving hormonal therapy, as well as identifying clinical and demographic risk factors associated with benign endometrial pathology and endometrial malignancy.

Clinical and demographic data were retrospectively extracted from the electronic medical records of 168 patients who met the inclusion criteria. Collected variables included demographic characteristics, clinical presentation, ultrasonographic findings, hysteroscopic findings, histopathological results, and pertinent medical history. All ultrasound examinations were performed using the GE LOGIQ E10s ultrasound system (GE Healthcare, Seoul, South Korea) by experienced radiologists. Transvaginal ultrasound (TVUS) was performed using a high-frequency endovaginal transducer (≥7.5 MHz). Endometrial thickness was measured in the sagittal plane at the thickest point of the endometrial echo, inclusive of both layers, in accordance with international sonographic guidelines. Seventy-six patients underwent TVUS. All patients were offered TVUS; however, depending on patient acceptability, body habitus, and clinical feasibility, the transabdominal acan (TAS) was performed in 92 patients.

It is acknowledged that TAS is associated with lower spatial resolution compared to TVUS for endometrial assessment. For the purposes of this analysis, measurements obtained by both modalities were combined into a single dataset, as the primary aim was to evaluate endometrial thickness as a diagnostic parameter regardless of the ultrasound route used. A subgroup analysis comparing diagnostic performance between TVUS and TAS was not performed due to sample size constraints.

For the purposes of diagnostic accuracy analysis, a positive ultrasound result was defined as an endometrial thickness of ≥5 mm in postmenopausal women, in accordance with established international thresholds. Hysteroscopy was performed by trained gynecologists. As it is a retrospective study, the doctors performing the hysteroscopy were not blinded to ultrasound findings. On hysteroscopy, a positive test result was defined as the presence of an abnormal-appearing endometrium, including irregular or polypoid surface architecture, abnormal vascularization, or focal lesions suspicious for malignancy, as assessed by the operating gynecologist at the time of the procedure.

Endometrial thickness measurements were categorized into the following predefined groups for analysis: <5 mm, 5-10 mm, 10.1-15 mm, 15.1-20 mm, and >20 mm. Histopathological findings were classified as either malignant (endometrial carcinoma) or benign (including endometrial polyps, hyperplasia without atypia, atrophic endometrium, and insufficient sample). Tissue specimens were obtained via a hysteroscopy-directed biopsy. Histopathological examination was used as the reference (gold) standard against which all diagnostic performance metrics were calculated.

Statistical analyses were performed using IBM SPSS Statistics version 25.0 (IBM Corporation, Armonk, NY, USA), and a p-value of <0.05 was considered statistically significant. Chi-square tests were employed to evaluate associations between categorical variables, with statistical significance defined as p <0.05. Sensitivity, specificity, PPV, and NPV were calculated with 95% confidence intervals (CIs) using standard 2×2 contingency tables, with histopathology as the reference standard. Only patients with complete data were taken for analysis.

## Results

The study cohort comprised 168 postmenopausal women with a mean age of 59.39 ± 7.83 years (range: 44-86 years). The mean body mass index (BMI) was 34.60 ± 7.60 kg/m² (range: 14.67-69.00 kg/m²), and the mean duration since menopause was 8.22 ± 6.66 years. Detailed demographic and clinical characteristics of the study population are presented in Table 1.

**Table 1:** Demographic findings P-value is for the association between the variable and endometrial cancer diagnosis. PCOS: polycystic ovary syndrome

Demographic findings	Categories	Endometrial Cancer N (%)	Benign Histopathology N (%)	P-value
Age	41 to 45 years	0	4(100%)	0.49
	46 to 50 years	0	13(100%)	
	51 to 55 years	1(2.5%)	39(97.5%)	
	56 to 60 years	2(4.7%)	41(95.3%)	
	61 to 65 years	3(10.0%)	27(90.0%)	
	>65 years	4(10.5%)	34(89.5%)	
BMI	Normal	0	6(100%)	0.60
	Overweight	1(2.6%)	38(97.4%)	
	Obese 1	3(5.7%)	50(94.3%)	
	Obese 2	4(10.8%)	33(89.2%)	
	Obese 3	2(6.1%)	31(93.9%)	
Parity	Nullipara	1(7.1%)	13(92.9%)	0.39
	Para	7(8.1%)	79(91.9%)	
	Grand multipara	2(2.9%)	66(97.1%)	
Risk factors	Absent	2(2.5%)	78(97.5%)	0.545
	PCOS	0	1(100%)	
	Infertility	0	2(100%)	
	Hypertension	3(11.5%)	23(88.5%)	
	Diabetes Mellitus	2(8.0%)	23(92.0%)	
	Hypertension and Diabetes	3(8.8%)	31(91.2%)	
Years since menopause	<10 years	5(4.7%)	101(95.3%)	0.463
	10 to 20 years	3(6.5%)	43(93.5%)	
	>20 years	2(12.5%)	14(87.5%)	

Traditional risk factors demonstrated no significant associations for endometrial malignancy, as detailed in Table 1, which could be due to a small sample size. Postmenopausal bleeding was the presenting symptom in 132 patients (78.6%), while only 3 patients (1.8%) were receiving hormone replacement therapy at the time of presentation. The majority of patients with postmenopausal bleeding exhibited endometrial thickness measurements between 5 and 10 mm.

In our cohort, the prevalence of metabolic and cardiovascular comorbidities among postmenopausal women with endometrial pathology was as follows: hypertension (11.5%), diabetes mellitus (8.0%), and both conditions concurrently (8.8%). These risk factors were present in 50.6% of benign cases and 80.0% of endometrial cancer cases.

No cases of endometrial carcinoma were identified among patients with endometrial thickness <5 mm. In contrast, endometrial malignancy was diagnosed in 5 of 11 patients (45.5%) with endometrial thickness >20 mm, demonstrating a statistically significant association (p < 0.005). The frequency and distribution of endometrial thickness stratified by histopathological diagnosis are presented in Table 2.

**Table 2:** Relationship of endometrial thickness with histopathology

Endometrial thickness	Frequency N (%)	Endometrial Cancer	Benign Histopathology	P-value
<5 mm	19 (11.3%)	0	19 (100%)	<0.0005
5-10 mm	72 (42.9%)	3 (4.2%)	69 (95.8%)	
10.1-15 mm	46 (27.4%)	1 (2.2%)	45 (97.8%)	
15.1-20 mm	20 (11.9%)	1 (5.0%)	19 (95.0%)	
>20 mm	11 (6.5%)	5 (45.5%)	6 (54.5%)	

Diagnostic performance of ultrasonography

At an endometrial thickness threshold of <5 mm, transvaginal ultrasonography demonstrated a sensitivity of 100%, specificity of 12%, positive predictive value (PPV) of 67%, and negative predictive value (NPV) of 100% for detecting endometrial malignancy (Table 3).

**Table 3:** Diagnostic performance of ultrasonography and hysteroscopy

Modality (ET Threshold)	Sensitivity	Specificity	PPV	NPV
Ultrasonography (<5 mm)	100%	12.0%	67.0%	100%
Hysteroscopy	80.0%	96.2%	57.1%	98.7%

Diagnostic performance of hysteroscopy

Hysteroscopic examination revealed abnormal-appearing endometrium in 14 patients, of whom 8 (57.1%) were subsequently diagnosed with endometrial carcinoma on histopathological analysis. Among the 154 patients with benign-appearing endometrium on hysteroscopic evaluation, only 2 (1.3%) were found to have endometrial malignancy. The overall diagnostic performance of hysteroscopy demonstrated a sensitivity of 80%, specificity of 96.2%, PPV of 57.1%, and NPV of 98.7% for identifying endometrial cancer (Table 3).

**Figure 1:**
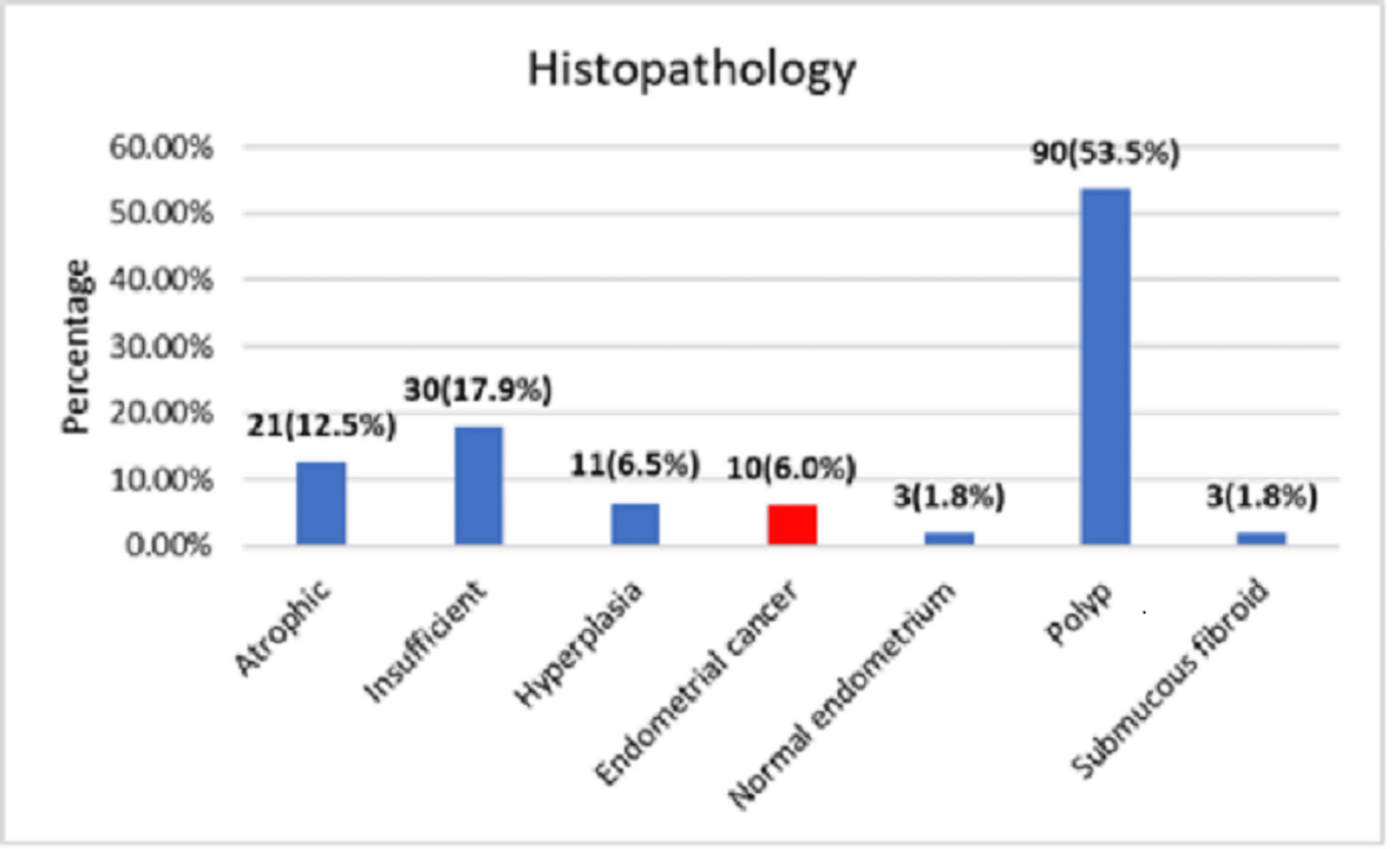
Histopathology findings

Histopathology findings showed that most of the patients had benign histopathology; 90 (53.5%) had benign polyps, and 51 (30.4%) had insufficient endometrium and atrophic endometrium (Figure 1). Eleven (6.5%) patients had endometrial hyperplasia, out of which 2 had atypical hyperplasia. Ten (6.0%) patients were diagnosed with endometrial cancer. One patient with complex hyperplasia had concomitant endometrioid adenocarcinoma.

In our study, 28 postmenopausal women had a history of breast cancer and underwent hysteroscopy due to a thickened endometrium with or without postmenopausal bleeding. Among them, 21 (75.0%) patients were receiving tamoxifen, while the remaining were on anastrozole or letrozole. Thirteen (46.4%) women presented with post-menopausal bleeding, while in a similar study, only 8% presented with vaginal bleeding and 88% were asymptomatic [12].

The mean ET in patients on tamoxifen was 12.05 +/- 5.2 mm. ET distribution revealed that 11 (52.4%) of the patients on tamoxifen had an ET between 10 and 15 mm, compared to 24.4% who had not received tamoxifen (P = 0.101). Nine (32.1%) of the patients had an ET of 5-10 mm, and 4 (14.3%) had an ET of 15-20 mm. While hysteroscopy revealed atrophic endometrium in 15 (53.6%) of cases and endometrial polyps in 11 (39.2%). Histopathological analysis revealed an insufficient sample and atrophic endometrium in 14 (50.0%), a polyp in 13( 46.4%), and normal endometrium in 1 (3.6%). No cases of endometrial cancer were identified in the cohort.

The correlation between ultrasonographic findings and histological outcomes in TAM-treated breast cancer patients has limitations. Most patients with an ET between 5 and 10 mm had an atrophic endometrial pattern on hysteroscopy, with insufficient samples obtained during histopathology, while patients with an ET >10 mm predominantly showed polyps on histopathological analysis. Despite the thickened endometrium in TAM-treated breast cancer patients, all of the histological findings were benign. Notably, none of the patients with a history of breast cancer had endometrial carcinoma.

## Discussion

Postmenopausal bleeding accounts for approximately 5% of gynecology clinic visits and necessitates prompt gynecologic evaluation to exclude endometrial malignancy. Patients presenting with postmenopausal bleeding carry a 10-15% risk of harboring endometrial carcinoma [7,10,15,16]. According to the American College of Obstetricians and Gynaecologists (ACOG) Committee Opinion, the risk of endometrial cancer increases progressively with advancing age [6]. In our cohort, among 10 patients diagnosed with endometrial carcinoma, 3 were aged 61-65 years and 4 were older than 65 years, findings consistent with previously published studies [17,18].

ACOG identifies nulliparity as an established risk factor for endometrial cancer. Our study population included 154 multiparous women, of whom 68 had parity ≥4, while only 14 women were nulliparous. Among patients with endometrial carcinoma, the majority were parous, with only one nulliparous patient. These findings are concordant with other studies reporting endometrial cancer prevalence of only 4-5% among nulliparous women [6,19,20].

The high prevalence of metabolic comorbidities in our cohort, particularly among those with endometrial cancer, reinforces the recognized association between these conditions and endometrial pathology. Notably, most of the patients with endometrial carcinoma were classified as obese, consistent with previously reported data [7-9,20,21].

Postmenopausal bleeding was the presenting symptom in 132 (78.6%) of our study population; among these patients, 8 were subsequently diagnosed with endometrial carcinoma, including 2 who experienced recurrent bleeding episodes. This aligns with established literature demonstrating that vaginal bleeding represents the presenting sign in more than 90.0% of postmenopausal women with endometrial carcinoma [6]. A comparable study reported that 40% of patients presented with a single bleeding episode, while 60.0% experienced recurrent bleeding [22].

In our series, 50% of patients with endometrial carcinoma had endometrial thickness >20 mm, while the remaining 50% demonstrated thickness between 5 and 20 mm. Importantly, no malignancies were identified in patients with endometrial thickness ≤5 mm. Beverly et al., in a systematic review and meta-analysis of 44 studies, demonstrated that endometrial thickness thresholds of 3 mm, 4 mm, or ≥5 mm yielded a pooled sensitivity of 96.2% and specificity of 51.5% for detecting endometrial cancer [23]. In our study, ultrasonography demonstrated 100% sensitivity for detecting endometrial malignancy at an endometrial thickness threshold of <5 mm, providing strong reassurance for ruling out cancer in this subset of patients.

Hysteroscopic findings were categorized as either abnormal-appearing or benign-appearing endometrium. Among 14 patients with abnormal-appearing endometrium on hysteroscopic examination, 8 (57.1%) were diagnosed with endometrial carcinoma on final histopathology, while 6 (42.9%) had benign pathology (simple or complex hyperplasia without atypia). Conversely, among 154 patients with a benign-appearing endometrium, only 2 (1.3%) were found to harbour underlying malignancy. The diagnostic performance of hysteroscopy in our study yielded a sensitivity of 80%, specificity of 96.2%, positive predictive value of 57.1%, and negative predictive value of 98.7%. These findings are comparable to those reported by Sousa et al., who documented a sensitivity of 97.7%, a specificity of 92.0%, a positive predictive value of 95.5%, and a negative predictive value of 95.8% [21]. The difference in sensitivity could be explained by different patient populations. Ultrasound specificity appears notably lower than in several prior studies; this could be attributed to differences in patient demographics and ET threshold.

**Table 4:** Comparison with similar studies Histopathology was used as the gold standard.

		Number	ET (mm)	USS				Hysteroscopy			
	Year			Sensitivity	Specificity	PPV	NPV	Sensitivity	Specificity	PPV	NPV
Our	2026	168	5	100%	12.0%	67.0%	100%	80%	96.2%	57.1%	98.7%
Garuti et al. [24]	1999	419	4	95.1%	54.8%	63.7%	-	96.5%	93.6%	92.6%	-
			8	83.8%	81.3%	79.4%	-				
Nisha et al. [25]	2019	100	4	86.4 %	42.3%	29.7%	91.7%	90.9 %	91.0 %	74.1 %	97.3%
Yela et al. [19]	2018	498 (postmenopausal)	5	99.0%	19.0%	96.1%	50.0%	96.7%	86.9%	99.2%	58.8%
Loiacono et al. [26]	2015	320	5	-	-	-	-	63.0%	97.0%	77.0%	95.0%

In our study, postmenopausal women had a history of breast cancer and underwent hysteroscopy; 13 (46.4%) women presented with post-menopausal bleeding, while in a similar study, only 8% presented with vaginal bleeding and 88% were asymptomatic [12]. Comparatively, Jindal et al. reported that 70.0% had an endometrial thickness of up to 5 mm, while 30.0% had an endometrial thickness of more than 5 mm. On hysteroscopy, 22.7% had abnormal hysteroscopic findings. Histopathological findings included secretory changes, polyps, atrophic endometrium, endometrial hyperplasia, endometrial adenocarcinoma, and scanty curetting [12].

In our cohort of breast cancer patients, the trend toward greater endometrial thickness in tamoxifen users, though not statistically significant, aligns with the known estrogen agonist effects of the drug on the endometrium. Despite the thickened endometrium observed on ultrasound, hysteroscopy frequently revealed atrophic changes, and all histopathological findings were benign. This discordance between ultrasound findings and final pathology underscores the poor specificity of TVUS in this population, as previously reported by Jindal et al. [12]. The absence of any endometrial malignancy in this high-risk subgroup is reassuring but should be interpreted with caution, given the small sample size.

Limitations

This study is subject to the inherent limitations of a single-center, retrospective design. Retrospective data collection introduces the possibility of selection bias, as only patients who had undergone ultrasound, hysteroscopy, and histopathological sampling were included, which may not be representative of the broader postmenopausal population presenting with abnormal uterine bleeding. Furthermore, the patient demographics, clinical protocols, and operator experience may differ substantially from those at other centres, thereby limiting the generalisability of our conclusions to wider clinical practice.

Another limitation of this study is the relatively small number of confirmed malignant cases (n=10), which restricts the statistical power of the diagnostic accuracy analyses and increases the width of the associated confidence intervals. This is of particular concern for subgroup analyses, including the breast cancer subgroup (n=28).

Methodological constraints are evident in the ultrasound's remarkably low specificity of 12%, which significantly undermines its reliability as a standalone diagnostic tool. This could be attributed to the use of transabdominal ultrasound in 92 cases. Furthermore, the high proportion of obese participants and the absence of long-term follow-up introduce additional uncertainties. While the research provides valuable insights into endometrial thickness and its relationship to pathology, particularly in postmenopausal and breast cancer populations, these limitations underscore the need for larger, more diverse, and longitudinal studies to validate and expand upon these findings. Further limitations, in the context of this retrospective study, concern the potential for observer bias arising from a lack of formal blinding and inter-observer variability in either ultrasound measurements or hysteroscopic interpretations.

Notwithstanding these limitations, this study provides clinically relevant preliminary data on the diagnostic performance of endometrial thickness measurement and hysteroscopy in the assessment of postmenopausal bleeding and contributes to the existing evidence base while identifying key areas for methodological refinement in future research.

## Conclusions

Postmenopausal bleeding represents a clinically significant symptom that warrants prompt and systematic evaluation to exclude endometrial malignancy. This study demonstrates that transvaginal ultrasonography serves as an effective first-line diagnostic modality for risk stratification based on endometrial thickness, exhibiting high sensitivity for detecting endometrial pathology. However, its limited specificity necessitates judicious interpretation to avoid overdiagnosis and unnecessary interventions. An endometrial thickness threshold of <5 mm reliably excludes malignancy, obviating the need for further invasive investigation in this low-risk subset.
